# Reading without spaces: The role of precise letter order

**DOI:** 10.3758/s13414-018-01648-6

**Published:** 2019-01-09

**Authors:** Jonathan Mirault, Joshua Snell, Jonathan Grainger

**Affiliations:** Laboratoire de Psychologie Cognitive, Centre National de la Recherche Scientifique, Aix-Marseille University, 3 place Victor Hugo, 13331 Marseille, France

**Keywords:** Sentence reading, Letter transpositions, Interword spacing, Reading aloud, Eye movements

## Abstract

Prior research points to efficient identification of embedded words as a key factor in facilitating the reading of text printed without spacing between words. Here we further tested the primary role of bottom-up word identification by altering this process with a letter transposition manipulation. In two experiments, we examined silent reading and reading aloud of normal sentences and sentences containing words with letter transpositions, in both normally spaced and unspaced conditions. We predicted that letter transpositions should be particularly harmful for reading unspaced text. In line with our prediction, the majority of our measures of reading fluency showed that unspaced text with letter transpositions was disproportionately difficult to read. These findings provide further support for the claim that reading text without between-word spacing relies principally on efficient bottom-up processing, enabling accurate word identification in the absence of visual cues to identify word boundaries.

## Introduction

A number of studies have investigated the ability of skilled readers to read text in which the extra interword spacing has been removed (e.g., Dreighe, Fitzsimmons & Liversedge, 2017; Epelboim, Booth, Ashkenazy, Taleghani & Steinman, 1997; Morris, Rayner & Pollatsek, 1990; Perea & Acha, 2009; Rayner, Fischer, & Pollatsek, 1998; Veldre, Drieghe & Andrews, 2017). The results of this research indicate that reading unspaced text is slower by about 40–70% relative to reading normally spaced text (Rayner & Pollatsek, 1996; Rayner et al., 1998). Readers make shorter saccades accompanied by longer fixations and more regressions when reading unspaced text, and the effect of word frequency on fixation durations is greater with unspaced text (Rayner et al., 1998). Furthermore, given the overall shorter saccade lengths, initial landing positions are closer to the beginning of words in unspaced text (Paterson & Jordan, 2010; Perea & Acha, 2009).^1^

The conclusion that has emerged from this research is that removing the spacing between words disrupts two distinct processes: saccade programming and word identification (Perea & Acha, 2009; Rayner et al., 1998). Firstly, given the key role for interword spaces in guiding eye movements during the reading of normally spaced text (e.g., Inhoff, Eiter, Radach, & Juhasz, 2003), removing interword spaces will logically affect saccade programming. The results of prior research suggest that readers adopt a more cautious oculomotor strategy when reading unspaced text, leading to a greater number of saccades per sentence (both forward and regressive) that are shorter in length. Secondly, the longer time spent inspecting each word when reading unspaced text (as reflected by longer fixation durations) is most likely due to the absence of visual cues for word beginnings and endings, and also possibly due to crowding effects occurring not only for the word’s inner-positioned letters, as is the case in normal (spaced) reading (e.g., Tydgat & Grainger, 2009), but also for the word’s outer-positioned letters.

In another study on reading unspaced text (Mirault, Snell, & Grainger, 2018) we investigated the role of sentence-level structures. In that study we compared reading of grammatically correct sentences and shuffled versions of the same words presented both with normal spacing and without spaces. In line with prior research, we found that reading was hampered by removing sentence structure (Schad, Nuthmann, & Engbert, 2010). Furthermore, there was only limited evidence that sentence structure facilitated the reading of unspaced text more so than reading spaced text. This pattern of results suggests that our ability to read grammatically correct unspaced text is not principally due to a greater involvement of top-down feedback from sentence-level structures.

On the other hand, our prior research did point to a key role for word identification processes in reading unspaced text, not only for linguistic processing, but also for guiding eye movements. We found that the length of the currently fixated word determined the amplitude of forward saccades leaving that word during the reading of unspaced text. This result suggests that readers of unspaced text use length information about the currently fixated word in order to program a saccade beyond that word’s rightward boundary. In the absence of visual cues, such length information can only be obtained by word identification providing access to information about word length. We therefore concluded that the relative ease with which skilled readers can read unspaced text is mainly due to efficient bottom-up word identification processes continuing to operate, and that support from sentence-level structures can facilitate these processes in certain conditions. Further support for this conclusion was found in the significantly greater impact of word frequency in the unspaced condition compared with normal spacing (see also, Veldre et al., 2017).

The present study was designed to further investigate the hypothesized importance of bottom-up word identification processes when reading unspaced text. Why might word identification be more important for reading unspaced text? First of all, we have shown that word identification guides eye movements when reading unspaced text, whereas the visual cues provided by interword spacing are the principal guiding factor when reading normally spaced text. Secondly, when reading unspaced text, word identification provides word order information that is necessary for the construction of a sentence-level representation. That is, the order in which words are identified is the main source of word order information, whereas with normally spaced text, the construction of a sentence-level representation benefits from the presence of interword spaces that facilitate the assignment of order information to word identities (Grainger, 2018; Snell & Grainger, 2017; Snell, Meeter, & Grainger, 2017). In line with this reasoning is the evidence obtained from readers of Thai, a language with an alphabetic script that does not use between-word spacing. It has been shown that Thai readers benefit from the artificial insertion of interword spaces, and the eye-movements of these readers suggest that this facilitation arises mainly at the level of word identification and sentence-level comprehension (Winskel, Perea, & Ratitamkul, 2012; Winskel, Radach, & Luksaneeyanawin, 2009).

In the present study, we tested the hypothesized greater role for word identification in reading unspaced text by selectively perturbing this process. We did so by introducing letter transpositions in certain words in each sentence. In a seminal study, Rayner, White, Johnson, and Liversedge (2006) recorded eye movements while participants read sentences that could either be formed of normally written words or contained a number of words with letter transpositions (e.g., The boy cuold not slove the probelm so he aksed for help). Rayner et al. reported that although reading text containing letter transpositions was relatively fluent, in line with prior findings from the single word recognition literature (e.g., Perea & Lupker, 2004; see Grainger, 2008, for a review), there was nonetheless a cost. That is, reading text containing letter transpositions induced longer fixation durations and more refixations and regressions compared with normally written text (see also Blythe, Johnson, Liversedge, & Rayner, 2014; White, Johnson, Liversedge, & Rayner, 2008).

The specific aim of the present study was to test the prediction that letter transpositions should have a significantly greater impact on reading unspaced text compared with normally spaced text. Two prior studies have conjointly manipulated between-word spacing and letter transpositions and have produced contradictory findings. Winskel et al. (2012) investigated the effects of letter transpositions and interword spacing in Thai. These authors reported an interfering effect of letter transpositions that did not interact with the spacing manipulation. However, the lack of an interaction in this study is likely due to the fact that Thai readers have developed efficient mechanisms for word segmentation in the absence of interword spacing, plus the fact that the presence of interword spaces is not natural for Thai readers. More directly related to the present study is the work of Johnson and Eisler (2012), who investigated effects of letter transpositions and interword spacing in English. The key results are those obtained in their Experiment 3, where rather than replacing interword spaces with filler stimuli, the typical greater spacing between words compared with inter-letter spacing was cancelled by increasing inter-letter spacing. The specific aim of that study, however, was to investigate effects of the position of letter transpositions, and the critical interaction between transposition effects (measured relative to a no-transposition condition) and spacing was not tested. Nevertheless, the condition means revealed much greater transposition effects in the absence of extra between-word spacing in all reading time measures except for first fixation durations. However, the choice to increase between-letter spacing rather than reducing between-word spacing might have impacted on their results. Therefore, in the present study we provide a further test of the predicted interaction between transposition effects and interword spacing in two experiments where normal inter-letter spacing was retained and the additional space between words was removed. In Experiment 1 we recorded eye movements while participants silently read sentences, and in Experiment 2 we collected audio recordings while participants read aloud the same set of sentences.

## Experiment 1: Silent reading

### Method

#### Participants

Thirty-two participants (24 female)^2^ from Aix-Marseille University, Marseille, France, received either €10 per hour or course credit for their participation. The participants were all native French speakers and gave written consent prior to the experiment. They reported having normal or corrected-to-normal vision, ranged in age from 18 to 28 years (M = 22.07, SD = 2.46), and were naïve with regard to the purpose of the experiment. French language skills were assessed using a Spelling Dictation test (Beyersmann, Casalis, Ziegler, & Grainger, 2015) and the LexTale vocabulary test (Brysbaert, 2013). Participants’ average scores were 78.59% (SD = 13.75) on the dictation test, and 88.37% (SD = 4.86) on the vocabulary test.

#### Design and stimuli

We constructed 104 sentences in French, each containing seven words. The sentences ranged in length from 37 to 57 characters including spaces (M = 47.94, SD = 3.77), and the average word frequency was 1,825 parts per million (ppm) (based on the Lexique2 film frequency counts: New, Pallier, Brysbaert, & Ferrand, 2004), which is equivalent to 6.26 Zipf (van Heuven, Mandera, Keuleers, & Brysbaert, 2014). Following a 2 × 2 factorial design, we manipulated between-word spacing (Spacing: spaced vs. unspaced) and word letter order (Transposition: normally written words vs. words containing transposed letters). The introduction of letter transpositions was constrained by five criteria: (i) the letters were adjacent consonants^3^, (ii) the first two and the last two letters of words were never transposed, (iii) the letters did not form a complex grapheme, (iv) the word containing the transposed letters was at least five letters long, and (v) words containing the transposed letters were always at the second position (verb), the fourth position (noun), and the fifth position (adjective) in sentences (i.e., three critical words per sentence contained letter transpositions in the transposed-letter condition). These critical words had an average frequency of 4.55 Zipf and an average length of eight letters. Words containing letter transpositions were never repeated across the different sentences seen by a given participant. Sentences were presented in lower case, except for the initial uppercase letter, and only contained letters without accents (see Appendix for a complete list the sentences and their transposed-letter versions). A Latin-square design was used with four groups of participants to ensure that all sentences were tested in all four conditions, but were seen only once per participant. Therefore a given set of three critical words were seen normally written and written with letter transpositions in both the spaced and unspaced conditions but by different participants.

#### Apparatus

Stimuli were displayed using OpenSesame (Mathôt, Schreij & Theeuwes, 2012), with each sentence occupying a single line. Eye movements were recorded with an EyeLink 1000 system (SR Research, Mississauga, ON, Canada) with high spatial resolution (0.01°) and a sampling rate of 1,000 Hz. Viewing was binocular, but only the right eye was monitored. The sentences were displayed on a gamma-calibrated 20-in. ViewSonic CRT monitor with a refresh rate of 150 Hz and a screen resolution of 1,024 x 768 pixels (30 x 40 cm). Stimuli were presented in black (0.15 Cd/m^2^) on a gray background (21.70 Cd/m^2^). Participants were seated 86 cm from the monitor, such that 3.6 characters equaled approximately 1° of visual angle. A chin-rest and a forehead-rest were used to minimize head movements.

#### Procedure

**Fig. 1:**
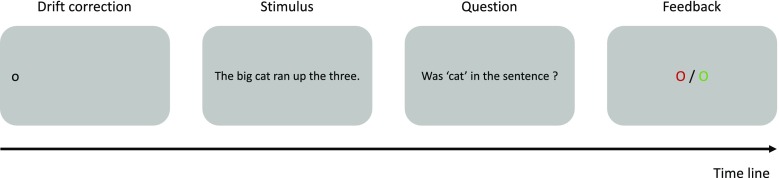
Trial procedure used in Experiment 1. Each trial started with a fixation dot located to the left of the display. When participants fixated the dot, a sentence was displayed. When the eye-position was determined to be beyond the sentence’s right end boundary, the sentence disappeared and the question display was presented until participants provided a button response. A feedback display was provided after each response

At the beginning of the experiment, the participant’s eye position was calibrated using a 9-point calibration grid. Each trial started with a drift correction dot located 200 pixels to the right of the left edge of the display (Fig. 1). Participants were instructed to focus on this dot, which would trigger the onset of a sentence stimulus. The distance between the fixation point and the beginning of the sentence was randomly determined, within a range of -54 to +32 pixels. Participants were instructed to read from left to right for comprehension. An invisible boundary was defined at the end of the sentence, such that the sentence disappeared when the eyes crossed that boundary. Next, participants were shown a question that allowed us to check whether they had paid attention to the word sequence. Participants were instructed to indicate whether they had seen a given word (e.g., “Did you see the word ‘table’?”) by means of a two-button response for, respectively, “yes” and “no” responses (probe word classification). Half of these questions concerned a word that was present in the sentence, and the other half a word that was not present in the sentence. The probe words never contained a letter transposition. Finally, a feedback dot was presented over 2,000 ms after the probe word classification response (green if the response was correct or red if the response was incorrect). The sentences were presented in a different random order for each participant. Participants received ten practice trials to familiarize them with the experimental procedure.

#### Analyses

We used linear mixed-effects models (LMEs) to analyze our data, with items and participants as crossed random effects (including by-item and by-participant random intercepts) (Baayen, Davidson, & Bates, 2008) and with random slopes (Barr, Levy, & Tily, 2013), and with Spacing and Transposition plus their interaction as fixed effects. The model successfully converged under this maximal random-effects structure in some but not all cases. In case of a failure to converge, we excluded the by-item random slopes (a Chi-square test indicated that a model including the by-item random slopes did not differ significantly from a model including the by-participant random slopes, so this was an arbitrary choice); and if a model then still failed to converge, we included only random intercepts. Generalized (logistic) linear mixed-effects models (GLMEs) were used to analyze the error rate and fixation probabilities. The models were fitted with the lmer (for LMEs) and glmer (for GLMEs) functions from the lme4 package (Bates, Maechler, Bolker, & Walker, 2015) in the R statistical computing environment. The condition with normal spacing and without letter transpositions was used as a reference and we reported regression coefficients (*b*), standard errors (SE),s and t-values (for LMEs) or z-values (for GLMEs) for all factors. Fixed effects were deemed reliable if |t| or |z| > 1.96 (Baayen, 2008). All duration measures were inverse-transformed (-1,000/duration) prior to analysis for the purpose of normalization.

### Results

The eye-movement data of one participant were removed prior to analysis due to a large number of eye blinks. All other participants depicted normal eye-movement behavior and responded with accuracy higher than 90% (M = 94.10, SD = 23.54) on the probe word classification trials. Response accuracy was significantly higher (*b* = 2.91; SE = 0.41; t = 6.97) with normally spaced sentences (98.47%) compared with unspaced sentences (89.75%). Prior to analysis we excluded trials containing blinks (5.04%) and trials with incorrect responses on the probe word classification task (5.89%). For the local word-based analyses, we used the data concerning the three critical target words in each sentence while excluding words that were skipped during first pass (1.81%). We measured and analyzed target word fixation durations and saccade type probabilities (skips, refixations, regressions), initial landing positions (ILPs; the location of the first forward fixation on a word), sentence reading speed, and estimated reading difficulty (evaluated by participants during post-experiment debriefing).

#### Fixation durations

**Fig. 2:**
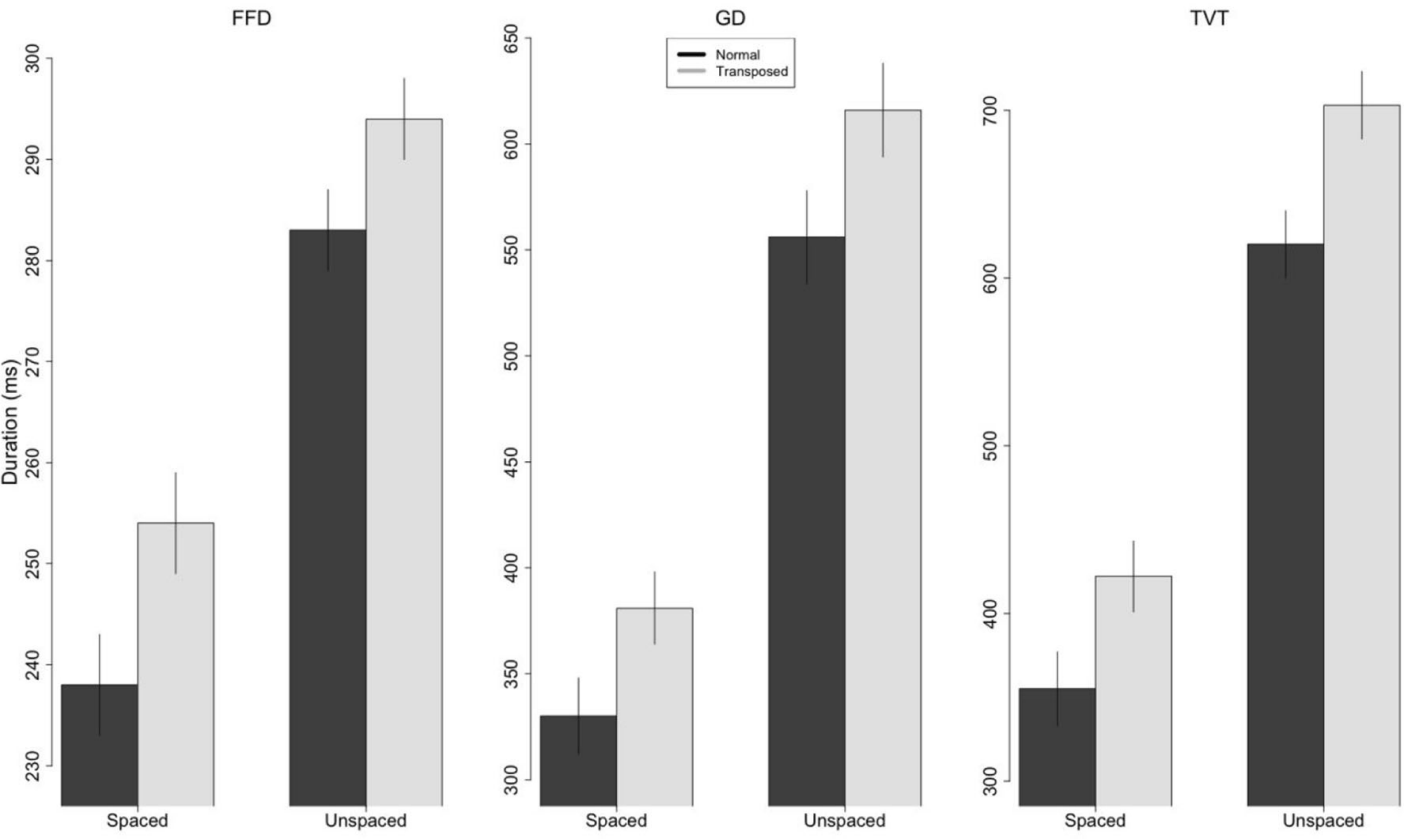
Average values (in ms) for fixation durations (*FFD* first fixation duration, *GD* gaze duration, *TVT* total viewing time) in Experiment 1. Error bars are the within-participants 95% confidence intervals (Cousineau, 2005). Y-axis scales are individually adapted to the different measures

From the eye-tracking data, we computed three fixation duration variables: First Fixation Duration (FFD), which represents the duration of the fixation immediately following the first forward saccade into a word; Gaze Duration (GD), which is the sum of all fixation durations on a word before the eyes leave that word (first pass fixations); and Total Viewing Time (TVT), which is the sum of all fixation durations on a word (thus including fixations made following a regressive saccade back to the word). These values were computed for the three critical target words in each sentence (i.e., words that involved a letter transposition manipulation in the transposition condition) and the average value per sentence entered in the analysis. From these data, we excluded words with values beyond 2.5 SD from the grand mean (FFD: 2.38%, GD: 2.46%, TVT: 2.98%). The mean duration values (in milliseconds) per experimental condition are presented in Fig. 2.

All the duration measures revealed significant effects of Spacing and Transposition, and in total viewing times there was also a significant interaction between these variables (see Table 1). Transposition effects were greater in the unspaced condition (83 ms) compared to the spaced condition (67 ms). We also analyzed second-pass reading times, which represent the amount of time spent re-reading a word after first-pass reading (Juhasz & Pollatsek, 2011). Here, we found a significant effect of Transposition (*b* = 0.10; SE = 0.03; t = **2.85**) with longer reading times in the transposed-letter condition, but neither the effects of Spacing (*b* = 0.02; SE = 0.05; t = 0.40) nor the interaction were significant (*b* = 0.09; SE = 0.05; t = 1.73).

**Table 1:** Fixed effects from the LMEs for the Fixation Duration measures in Experiment 1

	FFD			GD			TVT		
	*b*	SE	t	*b*	SE	t	*b*	SE	t
Spacing (S)	0.69	0.08	**8.29**	1.26	0.10	**12.35**	1.36	0.10	**12.64**
Transposition (T)	0.20	0.09	**2.28**	0.39	0.06	**5.95**	0.47	0.06	**7.34**
S x T	0.00	0.10	0.07	0.12	0.08	1.50	0.18	0.07	**2.45**

*Note.* Numbers in bold represent significant values

#### Saccade-type probabilities

**Fig. 3:**
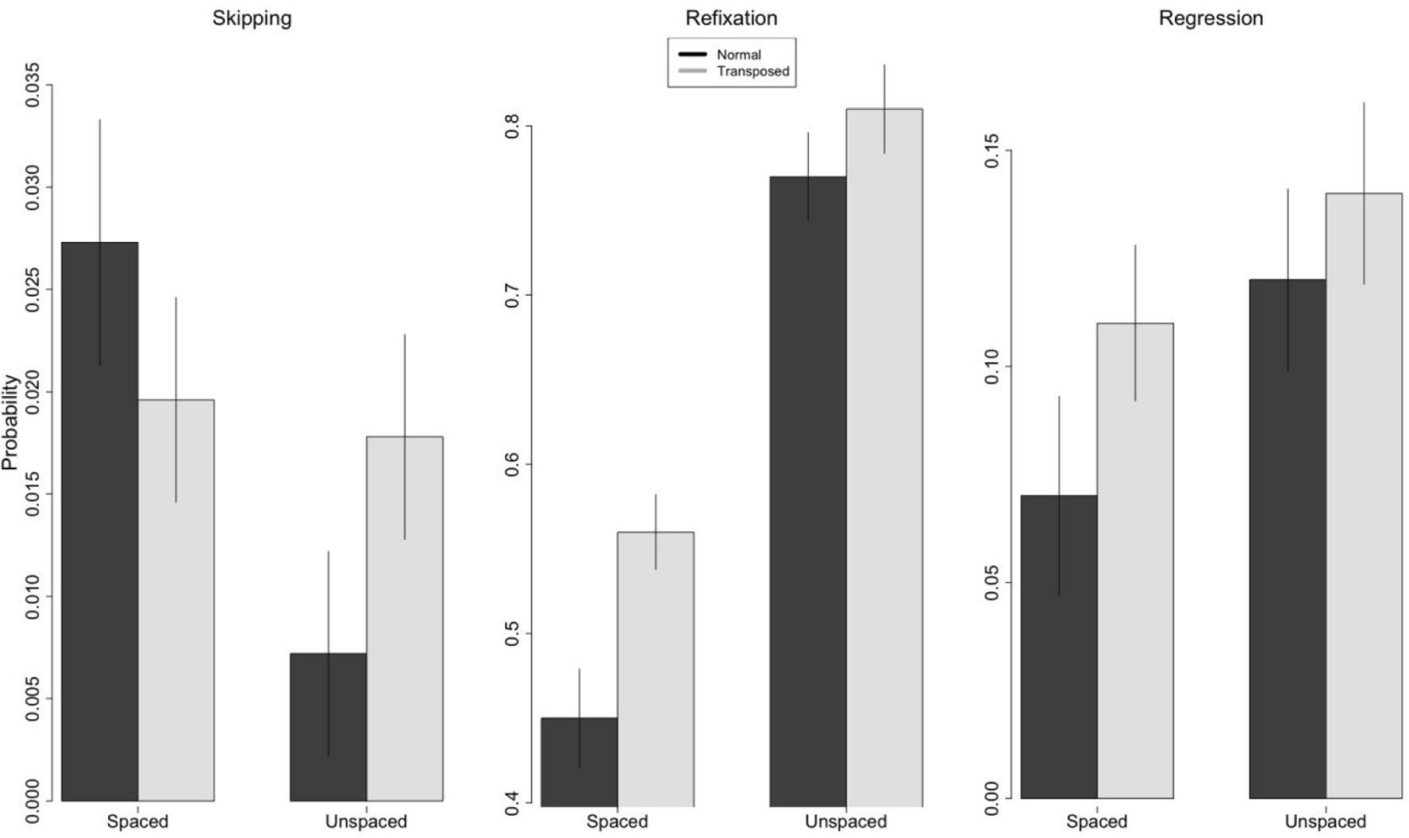
Average values for the different saccade type probabilities in Experiment 1. Error bars are the within-participants 95% confidence intervals (Cousineau, 2005). Y-axis scales are individialy adapted to the different measures

We calculated the probability of skipping a word (when a word is not fixated during first-pass forward eye movements), of refixating a word prior to leaving the word (within-word saccade), and of refixating a word after leaving that word (between-word regressive saccade). The average probabilities per experimental condition are shown in Fig. 3 and the results of the statistical analyses are reported in Table 2. We found that the absence of interword spaces caused a decrease in skipping probability accompanied by an increase in the probability of refixations and regressions. Letter transpositions had a significant effect in all three measures (Table 2), decreasing the skipping rate in the spaced condition and decreasing the skipping rate in the unspaced condition and increasing refixation and regression probabilities. For skipping probabilities, we observed an interaction between Spacing and Transposition, with a greater influence of letter transpositions in the unspaced condition compared to the spaced condition.

**Table 2:** Fixed effects from the GLMEs for the different measures of saccade type probabilities in Experiment 1

	SKIPPING			REFIXATION			REGRESSION		
	*b*	SE	z	*b*	SE	z	*b*	SE	z
Spacing (S)	1.25	0.37	**3.34**	1.62	0.14	**11.46**	0.28	0.21	1.29
Transposition (T)	0.35	0.21	1.66	0.48	0.06	**7.04**	0.47	0.11	**4.26**
S x T	1.22	0.38	**3.15**	0.10	0.11	0.90	0.27	0.15	1.80

*Note.* Numbers in bold represent significant values

#### Initial landing position (ILP)

**Fig. 4:**
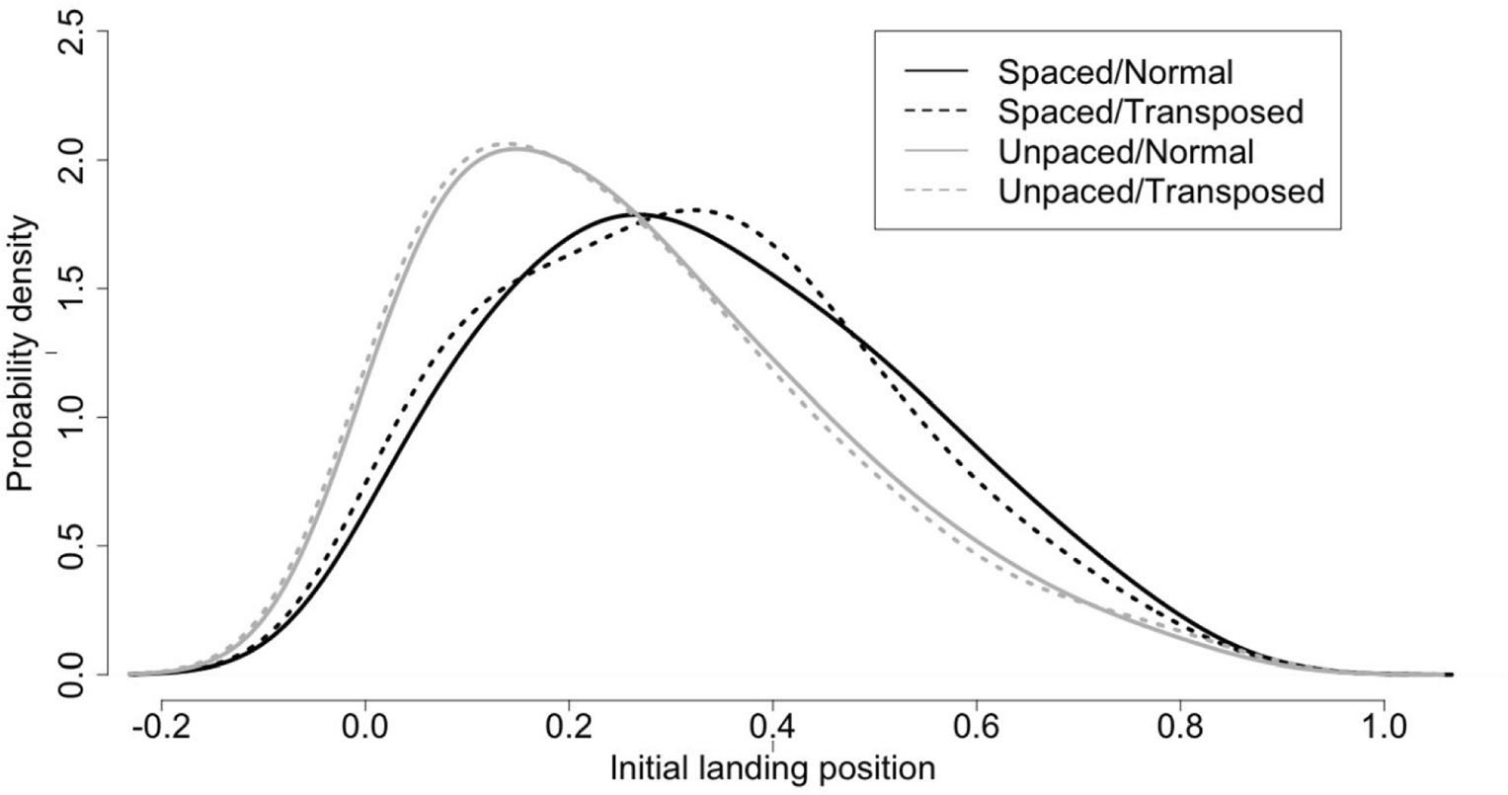
Distribution of initial landing positions in the four experimental conditions of Experiment 1. Curves represent the fitted Kernel density estimation. X-axis scale represents a normalized position between the beginning (0) and the end (1) of the word

Prior to statistical analysis of the initial landing positions (ILPs) we first excluded values lying beyond 2.5 SD from the mean (1.99%). Table 3 provides the mean ILP per experimental condition expressed in normalized values between the beginning (0) and the end (1) of words. The distributions of ILPs in each condition are shown in Fig. 4. There was a significant effect of Spacing (*b* = 0.06; SE = 0.00; t = **7.54**), with ILPs being closer to the beginning of words in the unspaced condition, and a significant effect of Transposition (*b* = 0.01; SE = 0.00; t = **2.04**), with the presence of transpositions causing the ILPs to shift slightly toward the beginning of words. The interaction between Spacing and Transposition was not significant (*b* = 0.00; SE = 0.00; t = 0.60).

**Table 3:** Mean initial landing positions from 0 (the beginning of the word) to 1 (the end of the word) in Experiment 1

		Transposition		
		Normal	Transposed	*TL effect*
Spacing	Spaced	.335 (0.008)	.321 (0.008)	*-.014*
	Unspaced	.263 (0.008)	.258 (0.008)	*-.005*

*Note.* 95% confidence intervals are given in parentheses

#### Sentence reading times

Sentence reading time was measured as the time between presentation of the stimulus and the moment participants’ eyes crossed the end boundary of the sentence. Thus, this measure gathered duration values for all the seven words of the sentence. Prior to analysis we excluded values beyond 2.5 SD from the mean (2.61% of trials). The average reading times (in ms) per experimental condition are shown in Table 4.

**Table 4:** Mean sentence reading times (ms) in the four experimental conditions of Experiment 1

		Transposition		
		Normal	Transposed	*TL effect*
Spacing	Spaced	1,875 (18.70)	2,107 (22.33)	*232*
	Unspaced	3,198 (31.36)	3,504 (35.60)	*306*

*Note.* 95% confidence intervals are given in parentheses

We found a significant effect of Spacing (*b* = 0.23; SE = 0.01; t = **16.72**) and Transposition (*b* = 0.05; SE = 0.01; t = **5.21**). The interaction between Spacing and Transposition was also significant (*b* = 0.02; SE = 0.01; t = **2.06**) with greater transposition effects in the unspaced condition compared to the spaced condition (see Table 4).

#### Estimated reading difficulty

In order to evaluate the subjective difficulty of reading in the different conditions, at the end of the experiment we asked participants to estimate their experienced reading difficulty in each condition. To do so, they were instructed to move a cursor on a scale from 0 to 100, and the corresponding number of the location of the cursor was always visible. Responses were collected without time limit, and no data were excluded prior to analysis. The average values for each condition are reported in Table 5.

**Table 5:** Mean of estimated reading difficulty on a scale from 0 (very easy) to 100 (very difficult) in Experiment 1

		Transposition		
		Normal	Transposed	*TL effect*
Spacing	Spaced	1.29 (1.00)	19.09 (5.16)	*17.80*
	Unspaced	29.58 (6.72)	68.67 (6.98)	*39.09*

*Note.* 95% confidence intervals are given in parentheses

We found significant effects of Spacing (*b* = 28.29; SE = 3.32; t = **8.51**) and Transposition (*b* = 17.80; SE = 2.60; t = **6.83**), and also a significant interaction (*b* = 21.29; SE = 3.68 t = **5.87**), with letter transpositions having a stronger effect when reading unspaced sentences compared to the normally spaced sentences (see Table 5).

#### Effects of vocabulary and spelling

Here we examined the impact of participants’ scores on the vocabulary and spelling dictation tests on the different dependent measures of Experiment 1, and whether these scores influenced the effects of Spacing and Transposition. We only report significant effects from LME and GLME analyses that successfully converged.

Gaze durations, total viewing times and initial landing positions were significantly influenced by vocabulary level (GD: *b* = 0.03; SE = 0.01; t = **2.05**; TVT: *b* = 0.04; SE = 0.01; t = **2.27**; ILP: *b* = 0.00; SE = 0.00; t = **2.62**), with higher vocabulary scores leading to shorter viewing durations and a shift of the ILP toward the middle of words. First fixation duration, gaze duration and total viewing times were significantly influenced by spelling ability (FFD: *b* = 0.01; SE = 0.00; t = **2.30**; GD: *b* = 0.01; SE = 0.00; t = **2.05**; TVT: *b* = 0.01; SE = 0.00; t = **1.98**), with higher spelling scores leading to shorter viewing durations.

More interesting is the fact that vocabulary level interacted with the effects of letter transpositions in gaze durations (*b* = 0.02; SE = 0.01; t = **1.98**), such that the interfering effect of transposing letters was greater in participants with higher vocabulary scores. Letter transposition effects also interacted with spelling ability in gaze durations (*b* = 0.00; SE = 0.00; t = **2.62**) and total viewing times (*b* = 0.01; SE = 0.00; t = **3.06**).

#### Effects of boundary letter frequency

In these analyses we report on the effects of boundary letter frequencies. Boundary letter frequency refers to the position-specific token frequency of the first and last letters in words. These analyses are motivated by the findings of Kasisopa, Reilly, Luksaneeyanawin, and Burnham (2013) showing an impact of such variables when reading in Thai and suggesting that these letter frequencies might act as a cue to word boundaries when reading unspaced text. Averages of the first letter and last letter frequency values (in Zipf) across the three critical words in each sentence were used in the LME and GLME analyses. First and last letter frequency were entered as separate variables given that Kasisopa et al. found more robust effects of these two variables when analyzed separately as opposed to a combined bigram frequency measure. Here we only report significant effects obtained in analyses that successfully converged.^4^

There was a significant three-way interaction involving first letter frequency in the gaze durations (*b* = 0.37; SE = 0.15; t = **2.32**) and total viewing times (*b* = 0.34; SE = 0.14; t = **2.42**). The two-way interaction between Spacing and Transposition (i.e., the greater effect of letter transpositions in the unspaced condition) was found to be stronger with low first letter frequencies (GD: *b* = 0.29; SE = 0.10; t = **2.78**; TVT: *b* = 0.32; SE = 0.09; t = **3.57**) compared to high first letter frequencies (GD: *b* = 0.00.; SE = 0.11; t = 0.00; TVT: *b* = 0.11; SE = 0.10; t = 1.03). There was also a significant three-way interaction involving last letter frequency in the total viewing times (*b* = 0.34; SE = 0.14; t = **2.42**). Again, the critical interaction between Spacing and Transposition was stronger when last letter frequency was low (*b* = 0.32; SE = 0.13; t = **2.46**) compared to high (*b* = 0.13; SE = 0.14; t = 0.96).

### Discussion

The results of Experiment 1 showed clear effects of both the Spacing factor and the Transposition factor on the majority of our measures of reading difficulty, both in terms of sentence-level measures (sentence reading speed and estimated reading difficulty), and in terms of local eye-movement behavior concerning the three critical target words in each sentence. The eye-movement results are in line with prior reports of effects of letter transpositions on fixation durations, and number of regressions and refixations (Blythe et al., 2014; Rayner et al., 2006; White et al., 2008), as well as prior reports of the influence of removing interword spaces on fixation durations, saccade-type probabilities, and initial landing positions (e.g., Mirault et al., 2018; Perea & Acha, 2009; Rayner et al., 1998). Crucial, with respect to the hypothesis under test, is that we observed a significantly stronger influence of letter transpositions when reading unspaced text compared with normally spaced text in total viewing times (per critical word) as well as for the sentence reading time and the estimated sentence reading difficulty. We also found that words containing letter transpositions were skipped more when reading unspaced text, whereas the opposite pattern was seen with normally spaced text. We return to discuss this finding in the *General discussion*. Overall, this pattern of results is in line with the hypothesized greater role for word identification processes when reading unspaced text, with letter transpositions selectively perturbing this process during reading.

In additional analyses we examined how the vocabulary scores and spelling ability of our participants influenced their reading behavior. The general pattern we observed was that higher vocabulary or spelling scores led to faster reading times in various measures. However, only vocabulary level affected initial landing positions, with a shift toward the middle of words for participants with higher scores. We also observed that the influence of vocabulary and spelling scores on certain duration measures was most pronounced in the condition with no letter transpositions. Vocabulary and spelling level had a much-reduced impact when reading text containing letter transpositions because participants with higher scores on these tests were more affected by interference from letter transpositions.

Finally, we found that differences in the frequency of the first and last letters of critical words impacted on the key interaction between Spacing and Transposition. The greater influence of letter transpositions in the unspaced condition significantly increased when first or last letter frequency was low. Low first and last letter frequencies increase uncertainty with respect to word boundaries, hence increasing the interference caused by introducing letter transpositions when there are no visual cues to word boundaries.

## Experiment 2: Reading aloud

Eye-movement recordings do not actually tell us if words are correctly identified in the different conditions, and more precisely, whether or not participants were actually identifying the basewords from which the transposed-letter stimuli were generated. Experiment 2 was therefore run in order to measure how well participants can actually identify words, including the basewords of transposed-letter stimuli, in the different experimental conditions. To do so, we asked participants to read aloud the same set of sentences as tested in Experiment 1, and we recorded the vocal output.

### Method

#### Participants

Twenty participants^5^ (12 females) following the same selection criteria as in Experiment 1. None of these participants had participated in Experiment 1. They ranged in age from 18 to 25 years (M = 21.6, SD = 2.22). Participants’ average scores were 63.04% (SD = 13.55) on the spelling dictation test, and 86.48% (SD = 3.77) on the vocabulary test.

#### Design and stimuli

We used the same design and the same stimuli as in Experiment 1.

#### Apparatus

Stimuli were created using OpenSesame (Mathôt et al., 2012) and displayed on a 15.5-in. LCD screen on a laptop computer. Sentences were presented in monospaced 18-point font in white (72.33 Cd/m^2^) on a gray background (63.61 Cd/m^2^). Participants were seated approximately 40 cm from the monitor, such that every two characters (0.7 cm) equaled approximately 1° of visual angle. We used an external microphone and Audacity to record the participants’ vocal responses. Uncompressed audio inputs were saved as .WAV files (32 bits).

#### Procedure

Instructions were first given orally, and then shown again on the screen before the experiment began. On each trial, first a dot centered on the screen was presented for 500 ms. Then a fixation cross was presented to the left (250 pixels from the center) and following that, the stimulus (a seven-word sentence) was shown for 4 s. Participants were instructed to read aloud the sentence from beginning to end. They were informed of the presence of letter transpositions (spelling mistakes) and instructed to try to read the corresponding word when they noticed such misspelled words. Vocal output was recorded for 4 s, and after that there was a short delay before the start of the next trial.

### Results

The data from one channel of the audio recordings was noise-filtered by first selecting a period of silence (for example a blank between two trials) to obtain the profile of the baseline noise frequency, and then removing that frequency band from the entire audio recording. The duration of each sentence produced by each participant was then manually measured, paying attention to exclude the breath artefact that occurred prior to articulation. Data concerning two participants were removed prior to analysis due to low scores on the spelling dictation test and high error rates in their reading aloud task. We measured reading speed and reading accuracy.

#### Reading speed

We measured reading speed in words per minute (wpm) for each sentence and each participant. Prior to analysis, we excluded values beyond 2.5 SD from the mean (< 1%). Means per condition are shown in Table 6.

**Table 6:** Mean reading speed (wpm) per condition in Experiment 2

		Transposition		
		Normal	Transposed	*TL effect*
Spacing	Spaced	156.84 (2.65)	133.11 (3.59)	*-23.73*
	Unspaced	103.08 (3.48)	72.43 (3.70)	*-30.65*

*Note.* 95% confidence intervals are given in parentheses

We found significant effects of Spacing (*b =* 84.88; SE = 4.1; t = **20.62**) and Transposition (*b* = 23.63; SE = 2.64; t = **8.93**). Reading aloud sentences took longer in the unspaced condition, and for sentences containing words with transposed-letters. We also found a significant interaction between these factors (*b* = 54.29; SE = 4.14; t = **13.11**), with a stronger influence of letter transpositions in the unspaced condition compared to the spaced condition (see Table 6).

#### Reading accuracy

The audio files obtained for each sentence and each participant were individually analyzed. We counted the number of correctly pronounced words in each sentence. We hand-coded as errors any word that was incorrectly pronounced (for example the word “maison” (*house* in English) could be incorrectly pronounced by addition of a phoneme (e.g., “marison”) or by substitution of a phoneme (e.g., “mason”)) or not pronounced either by omission or because the 4-s time-out had been reached. As concerns the words with letter transpositions, we counted as errors any pronunciation that did not correspond to the baseword, and here the most common error was the pronunciation of the transposed-letter version (i.e., a nonword), which strictly speaking is not an error, but was applied here in order to evaluate the extent to which words with letter transpositions were read aloud as the corresponding baseword. From this dataset, we calculated the percentage of trials with correct pronunciation per condition. We excluded values beyond 2.5 SD from the mean (6.51%). The results are summarized in Table 7.

**Table 7:** Mean percent correct pronunciations of target words/basewords per condition in Experiment 2

		Transposition		
		Normal	Transposed	*TL effect*
Spacing	Spaced	98.83 (0.51)	94.29 (1.93)	*-4.54*
	Unspaced	85.33 (1.08)	69.81 (2.57)	*-15.52*

*Note.* 95% confidence intervals are given in parentheses

We found significant effects of Spacing (*b =* 30.55; SE = 2.04; t = **14.93**) and Transposition (*b* = 4.63; SE = 1.01; t = **4.56**). We also found a significant interaction between these factors (*b* = 22.32; SE = 1.49; t = **14.29**) with a stronger influence of letter transpositions in the unspaced condition compared to the spaced condition (see Table 7).

#### Effects of vocabulary and spelling

Here we examined the impact of participants’ scores on the vocabulary and the spelling dictation tests on the two dependent measures of Experiment 2, and whether these scores influenced the effects of Spacing and Transposition. We only report significant effects from LME and GLME analyses that successfully converged. There were significant interactions with the Spacing factor in the reading speed and reading accuracy measures for both vocabulary level (Speed: *b* = 0.91; SE = 0.39; t = **2.31**, Accuracy: *b* = 0.89; SE = 0.22; t = **4.00**) and spelling ability (Speed: *b* = 0.38; SE = 0.18; t = **2.04**, Accuracy: *b* = 0.34; SE = 0.10; t = **3.28**). An increase in vocabulary level and spelling ability resulted in faster and more accurate reading of unspaced text, but not of normally spaced text.

#### Effects of boundary letter frequency

There were no significant effects of boundary letter frequency and no interactions with Spacing or Transposition in either reading speed or reading accuracy.

### Discussion

The results of Experiment 2 are clear-cut. Reading aloud sentences was slower and more error-prone in the absence of interword spaces, and when some of the words contained letter transpositions. Most important, however, is that the presence of transposed-letter stimuli made reading aloud significantly harder when reading unspaced text compared with normally spaced text. Furthermore, participants with higher vocabulary and spelling skills were faster and more accurate in reading, but only for unspaced text.

## General discussion

In two experiments, we set out to test the hypothesized greater role for bottom-up word identification processes in reading unspaced text compared with text printed with default interword spacing. Experiment 1 recorded eye movements as participants silently read sentences, and Experiment 2 recorded participants’ vocal output as they read aloud sentences. The sentences could either be written normally or contain words with letter transpositions (the critical target words). In both experiments we found evidence that the presence of letter transpositions had a greater negative impact on reading unspaced text compared with normally spaced text. This is in line with prior findings in English obtained in conditions where, rather than reducing inter-word spacing, inter-letter spacing was increased to match that of inter-word spacing (Johnson & Eisler, 2012). The interaction between the spacing manipulation and the presence vs. absence of letter transpositions was seen in the total viewing times and skipping rates for the critical target words, as well as in overall sentence reading times and participants’ self-evaluated reading difficulty in Experiment 1, and in reading aloud speed and accuracy in Experiment 2.

We interpret these findings as reflecting a greater reliance on bottom-up word identification processes during the reading of unspaced text compared with normally spaced text. Our letter transposition manipulation was specifically designed to perturb bottom-up word identification processes, and in line with prior research (e.g., Blythe et al., 2014; Rayner et al., 2006; White et al., 2008), we indeed found that reading normally spaced text with transposed letters was harder, inducing longer fixation durations, fewer skipped words and more within-word refixations and between-word regressions. We also reported, for the first time, that reading aloud of normally spaced sentences was harder in the presence of letter transpositions. The reading aloud data provided a more direct measure of word identification difficulty compared with eye-movement measures. The key finding of the present study is, however, that several measures of reading difficulty showed that this increased difficulty in reading sentences containing words with transposed letters was significantly greater in the absence of extra between-word spacing. It is this specific finding that points to a greater reliance on bottom-up word identification when reading unspaced text compared to normally spaced text.

In line with this interpretation of the present results is the finding, in Experiment 1, that the position-specific frequency of the initial and final letters of words impacted on the critical interaction between our spacing manipulation and the effect of transposed-letters. This interaction was found to be stronger when either first or last letter frequency was low. Following Kasisopa et al. (2013), we interpret this influence of boundary letter frequency as reflecting the use of such information for detecting word boundaries when reading unspaced text. Low letter frequency would make it harder to detect word boundaries, hence further exaggerating the impact of letter transpositions in the unspaced condition. Furthermore, we found that participants’ vocabulary level and spelling ability had a greater influence on the speed and accuracy with which they read aloud unspaced text compared to normally spaced text in Experiment 2. Veldre et al. (2017) had previously reported that spelling ability selectively influences the ability to read unspaced text, although they did not find a similar selectivity for their measure of reading ability. In spite of this minor divergence in the results, we agree with Veldre et al. that such findings point to better word identification skills having a particularly strong impact on the reading of unspaced text.

The results of Experiment 1 fit well with current models of eye movements and reading, such as EZ-Reader (Reichle, Pollatsek, Fischer, & Rayner, 1998), SWIFT (Engbert, Nuthmann, Ritcher, & Kliegl, 2005), Glenmore (Reilly & Radach, 2006), and OB1-Reader (Snell, van Leipsig, Grainger, & Meeter, 2018), which draw a clear distinction between decisions of where to move the eyes and decisions when to move the eyes. It is only the latter that are thought to be under cognitive control, and therefore modifiable by the cognitive processes involved in word identification, for example. Decisions where to move the eyes, on the other hand, would be mostly governed by low-level visual factors, and in particular by the information provided by between-word spaces when this is available. When this information is not available, then we suggest that readers resort to using word identification not only for making decisions about when to move the eyes, but also in deciding where to move the eyes. This would be combined with the more general strategy of making a greater number of shorter saccades when reading unspaced text. In line with this general strategy, we observed the typical pattern of a reduced skipping rate when reading unspaced text. However, we also found that there was an increase in skipping rate for words containing letter transpositions in the unspaced text condition. We very tentatively suggest that this might be due to an increased uncertainty in estimating where the next word lies, possibly with the transposed-letters being mistakenly used as cues for a word boundary.

The findings of the present study raise the issue as to exactly how word identification operates in the absence of extra between-word spacing. How are we able to identify written words when there are no visual cues to word boundaries? One account of orthographic processing is particularly easy to adapt to conditions where word beginning and ending information is absent. This is the family of models that use letter combinations to encode letter order (e.g., Dehaene, Cohen, Sigman & Vinckier, 2005; Mozer, 1987; Grainger & van Heuven, 2003; Whitney, 2001). These models do not require information about the beginning and ends of words in order to operate, but they can use between-word spaces as an additional source of positional information by combining spaces with letters (so-called “edge bigrams”). Furthermore, Grainger, Mathôt, and Vitu (2014) proposed that when reading normally spaced text, between-word spaces are used to limit the formation of ordered letter combinations to letters that appear within the same word. Therefore, when reading unspaced text, letter combinations would be formed both with letters from the same word and from letters belonging to different words. The interference caused by the generation of these inappropriate bigrams could be limited, however, by (1) limiting the inter-letter distance for forming bigrams or by weighting bigram activation by distance; and (2) by the influence of visual acuity, crowding, and spatial attention giving priority to processing of the currently fixated word (Grainger, Dufau, & Ziegler, 2016; Snell et al., 2017; 2018).

The efficiency with which word identification can proceed in the absence of interword spaces is perhaps not that surprising given the existence of written languages such as Thai, that use an alphabetic script without extra between-word spacing. Furthermore, highly agglutinative languages, such as Turkish and Finnish, use compounding to create very long words that have an internal structure with a similar level of complexity as entire sentences in non-agglutinative languages. Concerning this last point, it is interesting to note the recent theoretical proposal of Grainger and Beyersmann (2017), who suggested that one major mechanism for segmenting morphologically complex words is the non-morphological process of embedded word activation. In other words, the segmentation of polymorphemic words would involve processing similar to what occurs during the reading of unspaced text. In line with this are findings showing activation of embedded words independently of their morphological relation with the embedding stimulus (e.g., Bowers, Davis, & Hanley, 2005; Snell, Grainger, & Declerck, 2018).

In conclusion, we have provided further evidence for a greater role for bottom-up word identification processes during the reading of unspaced text compared with normally spaced text. These findings align with the evidence that sentence-level constraints play only a limited role in facilitating the reading of unspaced text (Mirault et al., 2018). Although sentence-level constraints do influence reading unspaced text, they are not the principal reason for why reading unspaced text is relatively easy. It is efficient bottom-up orthographic processing and word identification in the absence of word boundary information that is the primary factor at play. Future research could further explore the mechanisms involved in reading unspaced text by comparing the influence of within-word letter transpositions and between-word letter transpositions. A model of orthographic processing that uses letter combinations that are limited by interword spaces when these are present (Grainger et al., 2014) predicts that between-word transpositions should have a greater negative impact on reading normally spaced text compared with unspaced text. That is, we should observe the exact opposite pattern to what was found with within-word transpositions in the present study.

## Appendix

**Table 8:** The 104 sentences tested in the present study shown here with normal spacing. Each sentence is presented first without letter transpositions followed by the version with letter transpositions in the critical target words (in bold here for expository purposes)

n°	Sentence	n°	Sentence
1	Annie **corne** un **carnet robuste** par erreur	53	Huguette **forme** une **fonctionnaire enceinte** chez elle
	Annie **conre** un **canret robutse** par erreur		Huguette **fomre** une **fontcionnaire enceitne** chez elle
2	Arthur **converti** une **cicatrice grotesque** en tatouage	54	Jean **formule** des **directives obscures** pour demain
	Arthur **convetri** une **cicartice gortesque** en tatouage		Jean **fomrule** des **diretcives obcsures** pour demain
3	Alexandra **courtise** un **conservateur charmant** par amour	55	Julie **explique** la **trajectoire orbitale** du soleil
	Alexandra **coutrise** un **conseravteur chramant** par amour		Julie **exlpique** la **trajetcoire obritale** du soleil
4	Agathe **connecte** un **assemblage trompeur** aux autres	56	Constance **retarde** la **fiesta nocturne** a demain
	Agathe **conncete** un **assembalge tropmeur** aux autres		Constance **retadre** la **fietsa noctunre** a demain
5	Billy **condamne** un **chargement tordu** aux ordures	57	Eric **reproche** au **documentaire scientifique** le cadrage
	Billy **condmane** un **charegment trodu** aux ordures		Eric **rerpoche** au **documetnaire scietnifique** le cadrage
6	Charlotte **concentre** les **membres farfelus** au milieu	58	Emily **reporte** le **discours dogmatique** par cœur
	Charlotte **cocnentre** les **mebmres frafelus** au milieu		Emily **repotre** le **dicsours domgatique** par cœur
7	Denise **compacte** un **baudrier cintrant** sans efforts	59	Jules **cogne** un **employeur provincial** plusieurs fois
	Denise **copmacte** un **baudirer citnrant** sans efforts		Jules **conge** un **emlpoyeur provicnial** plusieurs fois
8	Dimitri **sacrifie** un **festin complaisant** pour nous	60	Edouard **reproduit** des **larves disparues** en France
	Dimitri **sacirfie** un **fetsin compailsant** pour nous		Edouard **rerpoduit** des **lavres dipsarues** en france
9	Daniel **saigne** un **porcinet adulte** pour midi	61	Edmond **répertorie** les **fourneaux modernes** de boulangerie
	Daniel **sainge** un **porcniet adlute** pour midi		Edmond **répetrorie** les **founreaux modenres** de boulangerie
10	Elsa **accompagne** un **centurion chanceux** au dehors	62	Jeanne **plante** une **mandarine germanique** au jardin
	Elsa **accopmagne** un **cetnurion chanecux** au dehors		Jeanne **platne** une **madnarine gemranique** au jardin
11	Enzo **collecte** des **courgettes vertes** les jeudis	63	Jacob **signale** un **menteur particulier** aux juges
	Enzo **colletce** des **cougrettes vetres** les jeudis		Jacob **singale** un **metneur patriculier** aux juges
12	Gaspard **satisfait** une **pulsion intense** chez lui	64	Ludivine **charme** un **ministre nerveux** avec insistance
	Gaspard **satifsait** une **puslion intesne** chez lui		Ludivine **chamre** un **minisrte nevreux** avec insistance
13	Hugo **sauvegarde** une **section importante** de jeu	65	Manon **rapatrie** un **montagnard perdu** en alaska
	Hugo **sauvegadre** une **setcion impotrante** de jeu		Manon **rapartie** un **motnagnard pedru** en alaska
14	Johan **scinde** un **abricot verdâtre** en deux	66	Nathan **apporte** au **gangster impertinent** mille euros
	Johan **scidne** un **arbicot verdârte** en deux		Nathan **appotre** au **gangtser impetrinent** mille euros
15	Joshua **accoste** un **cargo consistant** sans soucis	67	Noemie **installe** une **marmite fonctionnelle** en cuivre
	Joshua **accotse** un **cagro consitsant** sans soucis		Noemie **intsalle** une **mamrite fontcionnelle** en cuivre
16	Oscar **catapulte** une **carcasse charnue** super loin	68	Oriane **recense** les **migrations hivernales** en Belgique
	Oscar **cataplute** une **cacrasse chanrue** super loin		Oriane **recesne** les **mirgations hivenrales** en belgique
17	Rodolphe **berne** un **carnivore adolescent** pour rire	69	Omar **recommande** des **pigments festifs** pour peindre
	Rodolphe **benre** un **canrivore adloecsent** pour rire		Omar **recommadne** des **pimgents fetsifs** pour peindre
18	Tristan **assiste** un **apprenti consciencieux** chaque jour	70	Thibault **referme** une **pinte disponible** de vin
	Tristan **assitse** un **appretni consciecnieux** chaque jour		Thibault **refemre** une **pitne dipsonible** de vin
19	Yasmina **arrondi** une **alliance ternie** avec précision	71	Thierry **rembourse** un **manteau nordique** trente dollars
	Yasmina **arrodni** une **alliacne tenrie** avec précision		Thierry **rembousre** un **matneau nodrique** trente dollars
20	Wilfried **asperge** un **capricorne agressif** avec vigueur	72	Walid **rencontre** une **marmotte gourmande** qui mange
	Wilfried **aspegre** un **capriconre argessif** avec vigueur		Walid **recnontre** une **mamrotte goumrande** qui mange
21	Alexandre **retrouve** un **tableau harmonieux** ce matin	73	Xavier **remplace** le **docteur pervers** pour harcèlement
	Alexandre **rertouve** un **talbeau hamronieux** ce matin		Xavier **remlpace** le **dotceur pevrers** pour harcèlement
22	Adeline **kidnappe** un **chevreau calme** sans raison	74	Julien **gouverne** un **hospice providentiel** avec brio
	Adeline **kindappe** un **cherveau camle** sans raison		Julien **gouvenre** un **hopsice providetniel** avec brio
23	Amandine **regarde** un **chardon sombre** avec ardeur	75	Nelson **soigne** un **chevreuil perdu** ce lundi
	Amandine **regadre** un **chadron somrbe** avec ardeur		Nelson **soinge** un **cherveuil pedru** ce lundi
24	Damien **abandonne** une **veste magnifique** par terre	76	Gilles **coince** ses **doigts charnus** par inadvertance
	Damien **abadnonne** une **vetse mangifique** par terre		Gilles **coicne** ses **doitgs chanrus** par inadvertance
25	Romane **renverse** un **chaudron rempli** de soupe	77	Harry **illustre** ses **livres actuels** de dessins
	Romane **renvesre** un **chaurdon remlpi** de soupe		Harry **illusrte** ses **lirves atcuels** de dessins
26	Ondine **affectionne** les **berlingots maigres** en sucre	78	Helene **implante** des **sondes contre** le cancer
	Ondine **affetcionne** les **belringots mairges** en sucre		Helene **imlpante** des **sodnes conrte** le cancer
27	Franck **gronde** un **observateur pourvu** de jumelles	79	Jonathan **patiente** une grande quinzaine de minute
	Franck **grodne** un **obsevrateur pouvru** de jumelles		Jonathan **patietne** une gradne quiznaine de minute
28	Henri **accable** un **redoublant pensif** en cours	80	Khaled **porte** un **turban propre** sur lui
	Henri **accalbe** un **redoulbant pesnif** en cours		Khaled **potre** un **tubran prorpe** sur lui
29	Mathieu **conte** une **histoire mystique** aux enfants	81	Leslie **poursuit** des **malfrats irlandais** en voiture
	Mathieu **cotne** une **hitsoire mytsique** aux enfants		Leslie **pousruit** des **maflrats irladnais** en voiture
30	Mathilde **raccourcit** des **herbes grimpantes** au couteau	82	Johanna **influence** des **magistrats ignobles** avant midi
	Mathilde **raccoucrit** des **hebres gripmantes** au couteau		Johanna **ifluecne** des **magisrtats ingobles** avant midi
31	Pascale **commence** une **escapade monumentale** cet hiver	83	Laura **justifie** son **absence incroyable** fort lamentablement
	Pascale **commecne** une **ecsapade monumetnale** cet hiver		Laura **jutsifie** son **asbence inrcoyable** fort lamentablement
32	Michel **inonde** un **espion industriel** de critiques	84	Lucie **ralentit** ses **ardeurs persistantes** pour lui
	Michel **inodne** un **epsion indusrtiel** de critiques		Lucie **raletnit** ses **adreurs pesristantes** pour lui
33	Pauline **capte** un **imposteur marginal** trop bizarre	85	Laure **rafraichit** un **digestif gourmand** pour midi
	Pauline **catpe** un **impotseur magrinal** trop bizarre		Laure **rarfaichit** un **digetsif goumrand** pour midi
34	Vladimir **stocke** des **germes hospitaliers** au labo	86	Laurent **compte** les **festivals resplendissants** en alsace
	Vladimir **stokce** des **gemres hopsitaliers** au labo		Laurent **comtpe** les **fetsivals resplednissants** en alsace
35	Warren **licencie** un **directeur discordant** ce matin	87	Marie **raconte** une **fiction poignante** aux enfants
	Warren **licecnie** un **diretceur discodrant** ce matin		Marie **racotne** une **fitcion poingante** aux enfants
36	Youri **renvoie** une **directive personnelle** pour louise	88	Marc **range** son **pendentif soigneusement** au coffre
	Youri **revnoie** une **diretcive pesronnelle** pour louise		Marc **ragne** son **pendetnif soingeusement** au coffre
37	Emma **identifie** un **esprit plaintif** sans erreur	89	Myriam **charcute** un **dindonneau pulpeux** pour diner
	Emma **idetnifie** un **esrpit plaitnif** sans erreur		Myriam **chacrute** un **didnonneau pupleux** pour diner
38	David **respire** du **magnésium sulfureux** de soude	90	Maxime **inscrit** son **patronyme personnel** au tableau
	David **repsire** du **mangésium suflureux** de soude		Maxime **insrcit** son **partonyme pesronnel** au tableau
39	Natacha **interpelle** le **serveur distrait** pour boire	91	Marine **simplifie** son **forfait internet** chez free
	Natacha **inteprelle** le **sevreur disrtait** pour boire		Marine **simlpifie** son **fofrait intenret** chez free
40	Fred **facture** une **intervention expresse** cent euros	92	Nathalie **songe** au **pauvre pasteur** chez lui
	Fred **fatcure** une **intervention exrpesse** cent euros		Nathalie **sogne** au **paurve patseur** chez lui
41	Fatima **distribue** des **tracts rectangulaires** devant nous	93	Diane **parcourt** une **distance olympique** ce matin
	Fatima **disrtibue** des **tratcs rectagnulaires** devant nous		Diane **pacrourt** une **distacne olypmique** ce matin
42	Isabelle **discerne** du **basalte mince** en haut	94	Eve **riposte** aux **embuscades nombreuses** en irak
	Isabelle **dicserne** du **basatle micne** en haut		Eve **ripotse** aux **embucsades nomrbeuses** en irak
43	Odette **change** une **calandre abjecte** pour lui	95	Norbert **rassemble** des **disjoncteurs perpendiculaires** aux ordures
	Odette **chagne** une **calanrde abjetce** pour lui		Norbert **rassemlbe** des **dijsontceurs perpedniculaires** aux ordures
44	Pascal **vante** un **exploit merveilleux** totalement faux	96	Geoffroy **engendre** un **conflit insignifiant** avec toi
	Pascal **vatne** un **exlpoit mevreilleux** totalement faux		Geoffroy **engenrde** un **conlfit insingifiant** avec toi
45	Ernest **remplie** une **gourde gigantesque** de soda	97	Odile **parvient** aux **endroits grandioses** de sicile
	Ernest **remlpie** une **goudre gigatnesque** de soda		Odile **pavrient** aux **enrdoits gradnioses** de sicile
46	Corentin **restitue** un **pantalon moderne** au magasin	98	Paul **souscrit** un **contrat garanti** sans frais
	Corentin **retsitue** un **patnalon modenre** au magasin		Paul **sousrcit** un **cornrtat garatni** sans frais
47	Benoit **revendique** son **journal hebdomadaire** au marchant	99	Pablo **commande** un **parapente normal** chez lui
	Benoit **revednique** son **jounral hedbomadaire** au marchant		Pablo **commadne** un **parapetne nomral** chez lui
48	Cindy **participe** aux **olympiades scandinaves** sans stupeur	100	Philippe **recommence** une **action perpétuelle** sans arrêt
	Cindy **patricipe** aux **olypmiades scadninaves** sans stupeur		Philippe **recommecne** une **atcion peprétuelle** sans arrêt
49	Coraly **reste** aux **vendanges automnales** par plaisir	101	Florence **filtre** les **ressortissants islandais** aux barrages
	Coraly **retse** aux **vednanges autonmales** par plaisir		Florence **filrte** les **ressotrissants isladnais** aux barrages
50	Candice **partage** des **champignons fermes** avec sophie	102	Gregory **forge** un **sabre splendide** au japon
	Candice **patrage** des **chapmignons femres** avec sophie		Gregory **fogre** un **sarbe splednide** au japon
51	Colette **restreint** un **faisceau discret** de laser	103	Karim **hydrate** un **scientifique hurlant** en asie
	Colette **retsreint** un **faicseau discret** de laser		Karim **hyrdate** un **scietnifique hulrant** en asie
52	Claudine **embrasse** un **normand fastueux** au Portugal	104	Louis **distingue** un **serpent mortel** au loin
	Claudine **emrbasse** un **nomrand fatsueux** au portugal		Louis **ditsingue** un **seprent motrel** au loin

## Footnotes

1 It is important to note that the above-cited studies and the present study investigated reading of text in which the interword spaces have been removed, and not text in which the spaces have been replaced by filler stimuli (e.g., Malt & Seamon, 1978; Sheridan, Reichle, & Reingold, 2016).

2 Brysbaert and Stevens (2018) recommend at least 1,600 data points per condition. With 32 participants and 78 items per condition (3 words X 26 sentences in the main analyses) we therefore largely exceeded their recommendation of minimal experimental power.

3 Except for two sentences where mistakenly the transposition involved a consonant and a vowel.

4 Analyses of skipping rates, refixations, and regressions failed to converge.

5 Under the criterion of Brysbaert and Stevens (2018), this experiment is underpowered. However, we would argue that more stable data are obtained from the whole sentence measures of the present experiment compared with the individual word measures used to estimate power by Brysbaert and Stevens. The confidence intervals and t/z values obtained in this experiment confirmed our intuitions.
